# 1015. A multicenter evaluation of antimicrobial utilization in hospitalized bone marrow transplant (BMT) and T-cell therapy (CAR-T) patients, and the impact on rates of *C. difficile* infection (CDI)

**DOI:** 10.1093/ofid/ofad500.046

**Published:** 2023-11-27

**Authors:** Jerod Nagel, Kimberly D Boeser, Cory Hale, Alyssa Harris, Rupali Jain, Mike Postelnick, Emily S Spivak

**Affiliations:** Michigan Medicine, Ann Arbor, MI; M Health Fairview, University of Minnesota Medical Center, Minneapolis, Minnesota; Penn State Health Milton S. Hershey Medical Center, Hershey, Pennsylvania; Vizient, Inc, Chicago, Illinois; University of Washington Medical Center-Montlake, Seattle, WA; Northwestern Medicine, Chicago, Illinois; University of Utah School of Medicine, Salt Lake City, UT

## Abstract

**Background:**

Benchmarking antimicrobial utilization through the National Healthcare Safety Network (NHSN) will be a CMS requirement in 2024. NHSN benchmarking is available for some hospitalized populations, but there are currently no benchmarking capabilities for BMT units. Additionally, there are no published manuscripts describing antimicrobial utilization in BMT hospitals.

Intra-Hospital BMT Antimicrobial Utilization

**Figure:**
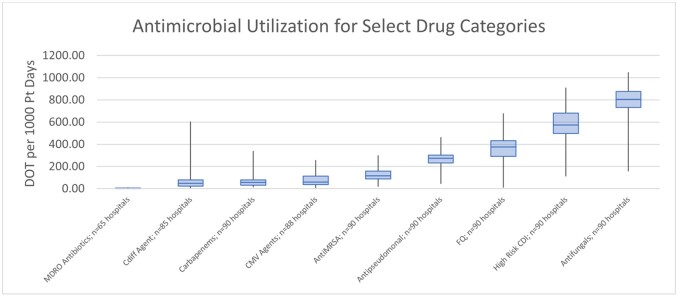

Data from the Vizient Clinical Data Base used with permission of Vizient, Inc. All rights reserved.

**Methods:**

This retrospective multicenter study was conducted by the Vizient Pharmacy Network AMS Committee. Antimicrobial utilization was evaluated for Vizient-member hospitals that treat at least 50 BMT patients per year. Vizient Clinical Database was used to identify patients via ICD10 codes for allogeneic transplant, autologous stem cell transplant and CAR-T therapy. Total antimicrobial days of therapy (DOTs) per 1,000 patient days was evaluated across all hospitals for each type of transplant, and for 9 different groups of antimicrobials (anti-pseudomonal beta-lactams, carbapenems, MDRO gram-negatives, fluoroquinolones, MRSA-active agents, antifungals, CMV-active agents, high-risk *C. difficile* agents, and *C. difficile* treatments). ANOVA was used to compare antimicrobial utilization across hospitals and across antimicrobial types.

**Results:**

A total of 90 hospitals and 63,379 patient encounters were included in the analysis from 2018-2021. The median total antimicrobial utilization for allogeneic, autologous, and CAR-T patients were 2,562, 2,083, and 2,187 DOTs per 1,000 patient days, respectively. Figure 1 describes the variability in intra-hospital prescribing for 9 antimicrobial categories. Hospitals with higher antimicrobial utilization rates in allogenic transplant patients were associated with a higher incidence of CDI in this population (p=0.0368).

**Conclusion:**

Significant variation in intra-hospital antimicrobial utilization exists for allogeneic transplant, autologous stem cell transplant, and CAR-T patients; and across 9 different categories of antimicrobials! Increasing rates of antimicrobial utilization were associated with increases in C. difficile infections in allogeneic transplants. Benchmarking antimicrobial utilization in BMT patients may help improve systematic approaches to minimize unnecessary antimicrobial exposure.

**Disclosures:**

**All Authors**: No reported disclosures

